# Junk food use and neurodevelopmental and growth outcomes in infants in low-resource settings

**DOI:** 10.3389/fpubh.2024.1308685

**Published:** 2024-04-15

**Authors:** Melody K. Chiwila, Nancy F. Krebs, Albert Manasyan, Elwyn Chomba, Musaku Mwenechanya, Manolo Mazariegos, Neelofar Sami, Omrana Pasha, Antoinette Tshefu, Adrien Lokangaka, Robert L. Goldenberg, Carl L. Bose, Marion Koso-Thomas, Norman Goco, Barbara T. Do, Elizabeth M. McClure, K. Michael Hambidge, Jamie E. Westcott, Waldemar A. Carlo

**Affiliations:** ^1^Global Network, University Teaching Hospital, Lusaka, Zambia; ^2^University of Colorado School of Medicine, Aurora, CO, United States; ^3^Department of Pediatrics, University of Alabama at Birmingham, Birmingham, AL, United States; ^4^Centre for Infectious Disease Research in Zambia, Lusaka, Zambia; ^5^Instituto de Nutrición de Centro América y Panamá, Guatemala City, Guatemala; ^6^Department of Community Health Sciences, Aga Khan University, Karachi, Pakistan; ^7^School of Public Health, University of Kinshasa, Kinshasa, Democratic Republic of Congo; ^8^Department of Obstetrics and Gynecology, Columbia University, New York, NY, United States; ^9^Department of Pediatrics, University of North Carolina, Chapel Hill, NC, United States; ^10^Eunice Kennedy Shiver National Institute of Child Health and Human Development, Bethesda, MD, United States; ^11^Research Triangle Institute International, Durham, NC, United States

**Keywords:** neonatology, infant nutrition, diet, low-income countries, complementary feeding, nutritional disorders, infant malnutrition, infant growth and development

## Abstract

**Introduction:**

Feeding infants a sub-optimal diet deprives them of critical nutrients for their physical and cognitive development. The objective of this study is to describe the intake of foods of low nutritional value (junk foods) and identify the association with growth and developmental outcomes in infants up to 18 months in low-resource settings.

**Methods:**

This is a secondary analysis of data from an iron-rich complementary foods (meat versus fortified cereal) randomized clinical trial on nutrition conducted in low-resource settings in four low- and middle-income countries (Democratic Republic of the Congo, Guatemala, Pakistan, and Zambia). Mothers in both study arms received nutritional messages on the importance of exclusive breastfeeding up to 6 months with continued breastfeeding up to at least 12 months. This study was designed to identify the socio-demographic predictors of feeding infants’ complementary foods of low nutritional value (junk foods) and to assess the associations between prevalence of junk food use with neurodevelopment (assessed with the Bayley Scales of Infant Development II) and growth at 18 months.

**Results:**

1,231 infants were enrolled, and 1,062 (86%) completed the study. Junk food feeding was more common in Guatemala, Pakistan, and Zambia than in the Democratic Republic of Congo. 7% of the infants were fed junk foods at 6 months which increased to 70% at 12 months. Non-exclusive breastfeeding at 6 months, higher maternal body mass index, more years of maternal and paternal education, and higher socioeconomic status were associated with feeding junk food. Prevalence of junk foods use was not associated with adverse neurodevelopmental or growth outcomes.

**Conclusion:**

The frequency of consumption of junk food was high in these low-resource settings but was not associated with adverse neurodevelopment or growth over the study period.

## 1 Introduction

Nutritionally appropriate foods are critical for optimal growth and development particularly during the first 2 years of life but evidence on the impact of nutritional deficiencies is limited (1). As infants grow, nutritional requirements increase and breastfeeding becomes insufficient, requiring the introduction of complementary foods starting at about 6 months (2), which may be important for infants’ health and cognitive development. Exclusive breast feeding up to 3 versus 6 months does not adversely affect neurodevelopment (3, 4). However, environmental, cultural, social, and economic factors can be associated with inappropriate complementary feeding practices (5–8). The low diversity of complementary foods may primarily comprise cereals, starch foods, and tubers, which, unless fortified, contain inadequate levels of essential micronutrients, iron, and protein-rich foods that are critical for normal growth (1, 9, 10). Reliance on such staple foods and a limited variety of all foods can reduce the nutritional intake to less than 50% of recommended values (11, 12). In a cross-sectional study of 1,500 infants conducted in low-resource settings, only 25% of infants received a diet containing animal protein, and no meat consumption was associated with stunting (5). However, in a randomized clinical trial of meat versus fortified cereal complementary feeds, linear growth was not affected and higher maternal education was associated with better linear growth (13). Additionally, maternal recall of feeding messages delivered in the trial was associated with linear growth (14). Other factors that affect feeding behaviors include family members, parity, and community, which also influence the selection of complementary feedings (15).

To address nutritional deficiencies during infancy, complementary feeding practices from 6 months onwards should include provision of adequate quantity, frequency, consistency, and variety of foods to cover the nutritional needs of the growing child while maintaining breastfeeding (1, 16). The European Society for Paediatric Gastroenterology, Hepatology, and Nutrition Committee (ESPGHAN) guidelines on nutrition indicate that infants should receive appropriate complementary feeds including iron-rich meat products and/or iron-fortified foods without sugar or salt added while avoiding fruit juices and sugar-sweetened beverages (17) but in low-resource settings, the frequent use of non-nutritious foods do not meet these needs (9, 18). Stunting is often irreversible and is associated with long term effects on health and neurodevelopmental capabilities of the child throughout his or her life (19, 20) including under-five mortality (21, 22). However, while there is substantial evidence on the effect of micronutrients, iron, iodine, caloric, and protein intake on short term nutrition (1), there are limited data on associations of low nutritional value food intake during infancy with growth and development.

Inexpensive packaged foods of low nutritional value are frequently given to infants as complementary foods. Because of their typical high palatability, these foods may be given to infants and young children instead of more nutritious complementary feeds. Certain infant and parent characteristics may be associated with increased use of junk foods (6–8, 10–15). Because of the uncertainty of the association of poor quality complementary feeds on infant outcomes (18–22), this analysis seeks to describe the intake of foods of low nutritional value (junk foods) and identify the association with growth and developmental outcomes in infants aged 6–18 months included in a complementary feeding randomized controlled trial in low-resource settings.

## 2 Methods

This study is a secondary analysis of a study entitled “Complementary Feeding: A Global Network Cluster Randomized Controlled Trial” (NCT01084109) conducted by the Global Network for Women’s and Children’s Health Research, a multi-country research network supported by the *Eunice Kennedy Shriver* National Institute of Child Health and Human Development (NICHD) (13). The trial was conducted in communities in the Democratic Republic of Congo, Guatemala, Pakistan, and Zambia. In a cluster randomized design, study participants were enrolled between 3 to 4 months of age after meeting the inclusion criteria of exclusive or predominantly breastfed with intention to continue breast-feeding through at least 12 months of life following parental consent. Exclusion criteria included receiving or likely to receive free or subsidized complementary foods, infant formula, or fortified foods, major congenital anomaly, infant of multiple births, or neurological deficit. Written informed consent for trial participation was obtained from the women or the parents/guardians of minors by trained staff. Once the study participants reached 6 months of age, freeze-dried beef was given to the intervention arm of infants and fortified rice-soy cereal to the control arm of infants as complementary feeds. Both study foods were delivered to the home on a weekly basis by research staff until the study participants reached 18 months of age. Mothers in both study arms were encouraged to continue breastfeeding with appropriate nutritional and health promotion messages on the importance of exclusive breastfeeding up to 6 months and continued breastfeeding up to at least 12 months; use of thickened porridges; feeding complementary foods 3 times per day; and feeding a variety of nutritious locally available complementary food from 6 to 18 months. Bias in data collection was reduced with the use of qualitative 24-h dietary recalls for the previous day collected without judgment on specifically designed data forms at 6, 9, 12 and 18 months.

Per the trial protocol (23), the Bayley Scales of Infant Development, 2nd Edition, (BSID II) was administered at 18 ± 1 months of age. Briefly, the BSID II was performed by trained and certified evaluators who received training from an experienced psychologist and Bayley II trainer. To avoid bias, the evaluators were masked to the intervention allocation. The evaluators administered the BSID II directly to each child in the local language. Mental Developmental Index (MDI) or Psychomotor Developmental Index (PDI) scores are reported.

This secondary analysis seeks to identify the predictors of feeding infants junk foods as part of complementary foods and the associations of prevalence of junk food intake with neurodevelopment and growth. Complementary foods of low nutritional value and high content of energy, fat, sugar, and/or salt that are highly processed were defined as junk foods (3). Junk foods were classified as cookies, candies, salty snacks, and sweet drinks. Standardized 24-h qualitative data on food consumption including junk food were collected by trained assessors from parental recall at 6, 9, 12, and 18 months of age.

Descriptive statistics were reported for baseline parental and neonatal characteristics. Socioeconomic status (SES) was estimated with a composite variable using economic indicators including dwelling, roof, wall, floor, toilet type, and water source to score the family’s relative wealth (an average SES score would be 0, below average < 0, and above average > 0) (14). Frequencies and percentages were reported for categorical variables with differences in characteristics tested for by chi-square. Means and standard deviations were reported for continuous variables with difference in mean tested using Student’s t-test. Further, junk food feeding practices were reported by postnatal age and country (DRC, Guatemala, Pakistan, and Zambia). Adjusted relative risks (RR) and 95% confidence intervals (CIs) for qualitative junk food consumption at 18 months were estimated using Poisson regression models including potential predictors of SES, sex, exclusively breast fed for 6 months, birth weight, preterm status, vitamin supplementation, maternal BMI, maternal and paternal education level, and number of living children while controlling for site and intervention group. Tests of interaction of each potential predictor with site were conducted to assess whether the relationship between each measure and developmental outcome differed between the four sites; adjusted relative risks are shown by site if interactions were significant. In a similar fashion, linear regression models were used to assess the association between prevalence of junk food use in children at 9 and 12 months with anthropomorphic measures at 18 months controlling for site, intervention group, SES, sex, exclusively breast fed for first 6 months, birth weight, preterm status, maternal and paternal education level, and vitamin supplementation. Separate models were generated separately for each junk food type and each anthropomorphic measure. Data were entered at each site into local computers and transmitted to a central data coordinating center (RTI International, Durham, NC). Data were analyzed by using SAS statistical software (SAS Inc. v.9.4). Because of multiple analyses, a *p* value <0.01 was used as a cut-off for statistical significance.

## 3 Results

There were 1,231 enrolled infants in the primary trial (12). 1,062 (86%) had complete data on junk food use. The study sites in participating countries contributed almost equal number of participants. Parental and neonatal characteristics by site and overall are provided in Table 1. Briefly, the average weight of infants at birth was 3,163 ± 527 g; 51% were females; 50% were reported to have been exclusively breastfed at 6 months but with wide site variability from 1% in Pakistan to 96% in Zambia. Both maternal education and composite SES score were the lowest in DRC. Baseline differences in characteristics for infants who consumed junk food at 9 and 12 months compared to those who did not consume junk food included non-exclusive breastfeeding at 6 months, prematurity, higher maternal body mass index, more years of maternal and paternal education, higher SES scores, and vitamin supplementation (Table 2).

**Table 1:** Parental and neonatal characteristics by site.

	DRC *N* = 271	Zambia *N* = 246	Guatemala *N* = 267	Pakistan *N* = 278	Total *N* = 1,062
Study participant characteristics					
Birth weight (g), M ± SD	3,204 ± 482	3,266 ± 529	3,107 ± 483	3,053 ± 615	3,163 ± 527
Female, *n* (%)	132 (49)	123 (50)	136 (51)	147 (53)	538 (51)
Exclusively breastfed at 6 months, *n* (%)	247 (91)	236 (96)	46 (17)	3 (1)	532 (50)
Prematurity, *n* (%)	6 (2)	9 (4)	19 (7)	27 (10)	61 (6)
Vitamin supplements, *n* (%)	0 (0)	246 (100)	137 (51)	41 (15)	424 (40)
Parental characteristics					
Maternal BMI, M ± SD	20.7 ± 2.3	24.8 ± 4.4	24.4 ± 3.7	23.2 ± 4.7	23.2 ± 4.2
Number of living children, M ± SD	3.0 ± 1.8	3.5 ± 2.0	2.8 ± 2.1	2.8 ± 1.7	3.0 ± 1.9
1–2, *n* (%)	127 (47)	93 (38)	151 (57)	138 (50)	509 (48)
3–4, *n* (%)	89 (33)	80 (33)	74 (28)	96 (35)	339 (32)
≥5, *n* (%)	55 (20)	73 (30)	42 (16)	44 (16)	214 (20)
Mother employed	3 (1)	23 (9)	53 (20)	15 (5)	94 (9)
Maternal education (years), M ± SD	2.4 ± 2.7	6.4 ± 3.1	4.9 ± 3.6	6.4 ± 4.7	5.0 ± 4.0
0, *n* (%)	114 (42)	19 (8)	31 (12)	74 (27)	238 (22)
1–7, *n* (%)	143 (53)	151 (61)	174 (65)	60 (22)	528 (50)
≥8, *n* (%)	14 (5)	76 (31)	62 (23)	144 (52)	296 (28)
Paternal education (years), M ± SD	6.6 ± 3.3	7.9 ± 3.2	5.7 ± 4.0	7.4 ± 4.6	6.9 ± 3.9
0, *n* (%)	17 (6)	12 (5)	35 (13)	55 (20)	119 (11)
1–7, *n* (%)	152 (56)	117 (48)	149 (56)	49 (18)	467 (44)
≥8, *n* (%)	102 (38)	117 (48)	83 (31)	174 (63)	476 (45)
SES composite score, M ± SD	−4.1 ± 0.5	−0.3 ± 2.0	2.3 ± 1.1	1.8 ± 1.7	−0.04 ± 2.9
Low (≤−1.3), n (%)	269 (99)	72 (29)	0 (0)	11 (4)	352 (33)
Medium (−1.3 < SES < 2.8), n (%)	2 (1)	160 (65)	157 (59)	148 (53)	467 (44)
High (≥2.8), n (%)	0 (0)	14 (6)	110 (41)	119 (43)	243 (23)
MDI at 18 months, M ± SD	97.5 ± 10.2	99.2 ± 10.5	92.7 ± 8.7	93.4 ± 9.4	95.6 ± 10.0
PDI at 18 months, M ± SD	101.5 ± 11.7	102.2 ± 10.0	96.2 ± 11.8	101.2 ± 8.8	100.2 ± 10.9
Anthropometric measurements at 6 months					
Length Z-score, M ± SD	−1.72 ± 1.13	−0.79 ± 1.38	−2.23 ± 0.96	−0.70 ± 1.17	−1.37 ± 1.33
Weight Z-score, M ± SD	−1.24 ± 1.15	−0.41 ± 1.23	−0.88 ± 1.16	−1.21 ± 1.08	−0.95 ± 1.20
Weight-for-Length Z-score, M ± SD	−0.08 ± 1.25	0.24 ± 1.42	0.91 ± 0.98	−0.97 ± 1.27	0.01 ± 1.41
Head Circumference Z-score, M ± SD	−0.46 ± 1.14	0.20 ± 1.28	−0.64 ± 0.93	−0.65 ± 1.07	−0.40 ± 1.16
Stunting, *n* (%)	113 (42)	50 (20)	160 (60)	35 (13)	358 (34)
Wasting, *n* (%)	21 (8)	12 (5)	1 (0)	53 (19)	87 (8)

SES (Socioeconomic Status); MDI (Mental Development Index); PDI (Psychomotor Development Index).

**Table 2:** Parental and neonatal characteristics by junk food consumption by 9 and 12 months.

	Feeding habits by 9 months			Feeding habits by 12 months		
	Did not consume junk food	Consumed junk food	*p* value^†^	Did not consume junk food	Consumed junk food	*p* value^†^
Study participant characteristics						
Birth weight (g), M ± SD	3,173 ± 510	3,153 ± 545	0.20	3,193 ± 494	3,149 ± 542	0.03
Female, *n* (%)	260 (50)	278 (51)	0.59	156 (48)	382 (52)	0.34
Exclusively breastfed at 6 months, *n* (%)	322 (62)	210 (39)	<0.01	240 (75)	292 (39)	<0.01
Prematurity, *n* (%)	22 (4)	39 (7)	0.03	10 (3)	51 (7)	0.01
Vitamin supplements, *n* (%)	148 (28)	276 (51)	<0.01	53 (16)	371 (50)	<0.01
Parental characteristics						
Maternal BMI, M ± SD	22.7 ± 4.0	23.7 ± 4.4	<0.01	21.9 ± 3.5	23.8 ± 4.3	<0.01
Number of living children, M ± SD	3.0 ± 1.9	3.0 ± 2.0	0.22	3.0 ± 1.8	3.0 ± 2.0	0.34
1–2, *n* (%)	243 (47)	266 (49)	0.15	148 (46)	361 (49)	0.57
3–4, *n* (%)	181 (35)	158 (29)		110 (34)	229 (31)	
≥5, *n* (%)	98 (19)	116 (21)		64 (20)	150 (20)	
Mother employed, *n* (%)	38 (7)	56 (10)	0.08	14 (4)	80 (11)	<0.01
Maternal education (years), M ± SD	4.2 ± 3.9	5.8 ± 3.9	<0.01	3.4 ± 3.5	5.7 ± 4.0	<0.01
0, *n* (%)	145 (28)	93 (17)	<0.01	111 (34)	127 (17)	<0.01
1–7, *n* (%)	267 (51)	261 (48)		167 (52)	361 (49)	
≥8, n (%)	110 (21)	186 (34)		44 (14)	252 (34)	
Paternal education (years), M ± SD	6.8 ± 3.8	7.0 ± 4.0	0.19	6.6 ± 3.6	7.0 ± 4.1	0.02
0, *n* (%)	49 (9)	70 (13)	0.01	26 (8)	93 (13)	<0.01
1–7, *n* (%)	252 (48)	215 (40)		172 (53)	295 (40)	
≥8, *n* (%)	221 (42)	255 (47)		124 (39)	352 (48)	
SES composite score, M ± SD	−1.2 ± 3.1	1.0 ± 2.2	<0.01	−2.4 ± 2.9	1.0 ± 2.3	<0.01
Low (≤−1.3), *n* (%)	273 (52)	79 (15)	<0.01	236 (73)	116 (16)	<0.01
Medium (−1.3 < SES < 2.8), *n* (%)	157 (30)	310 (57)		47 (15)	420 (57)	
High (≥2.8), *n* (%)	92 (18)	151 (28)		39 (12)	204 (28)	

^†^Differences in categorical characteristics tested for by chi-square and differences in continuous variables tested using Student’s *t*-test.

SES (Socioeconomic Status).

Junk food feeding practices differed by age and site (Table 3). As a multicenter study, data on brands and homemade products were not available. Feeding junk food increased with increasing post-natal age. A minority of infants (7%) were fed any junk food as early as 6 months. The rate of feeding junk food increased to 51% at 9 months, 70% at 12 months, and 78% at 18 months. Intake of cookies, salty snacks, sweet drinks, and any junk food were substantially lower in DRC throughout the whole study period compared to all other sites but intake of candies did not differ by site. Zambia and Pakistan had earlier introduction of junk food. Feeding any junk food at 18 months was substantially lower in DRC (23%) and more common in Pakistan (99%), Zambia (98%), and Guatemala (95%). The most common category of junk food with the highest prevalence use at 18 months of age was cookies (61%) followed by salty snacks (51%) and sweet drinks (49%). The type of junk foods differed by sites with cookies and candies consumed the most in Pakistan whereas salty snacks and sweet drinks were most consumed in Zambia and Guatemala. In adjusted analyses, country was the strongest predictor of junk food intake (Table 4). After removal of the DRC data, the characteristics for infants who consumed junk food at 9 months did not differ compared to those who did not consume junk food (Table 5).

**Table 3:** Junk food feeding practices by age and site.

Type of Snack	DRC *N* = 271	Zambia *N* = 246	Guatemala *N* = 267	Pakistan *N* = 278	Total *N* = 1,062
Cookies, *n* (%)					
6 months	0 (0)	3 (1)	10 (4)	38 (14)	51 (5)
9 months	7 (3)	93 (38)	67 (25)	172 (62)	339 (32)
12 months	12 (4)	139 (57)	131 (49)	234 (84)	516 (49)
18 months	20 (7)	191 (78)	169 (63)	264 (95)	644 (61)
Candies, *n* (%)					
6 months	2 (1)	0 (0)	5 (2)	1 (0)	8 (1)
9 months	18 (7)	22 (9)	18 (7)	6 (2)	64 (6)
12 months	35 (13)	41 (17)	44 (16)	24 (9)	144 (14)
18 months	50 (18)	91 (37)	79 (30)	87 (31)	307 (29)
Salty snacks, *n* (%)					
6 months	0 (0)	0 (0)	1 (0)	4 (1)	5 (0)
9 months	0 (0)	102 (41)	13 (5)	78 (28)	193 (18)
12 months	0 (0)	180 (73)	37 (14)	146 (53)	363 (34)
18 months	0 (0)	231 (94)	101 (38)	206 (74)	538 (51)
Sweet drinks, *n* (%)					
6 months	1 (0)	0 (0)	14 (5)	0 (0)	15 (1)
9 months	1 (0)	103 (42)	99 (37)	15 (5)	218 (21)
12 months	2 (1)	172 (70)	173 (65)	32 (12)	379 (36)
18 months	4 (1)	221 (90)	220 (82)	80 (29)	525 (49)
Any junk food, *n* (%)					
6 months	2 (1)	3 (1)	27 (10)	40 (14)	72 (7)
9 months	22 (8)	173 (70)	144 (54)	201 (72)	540 (51)
12 months	40 (15)	224 (91)	217 (81)	259 (93)	740 (70)
18 months	62 (23)	241 (98)	254 (95)	275 (99)	832 (78)

**Table 4:** Potential predictors for junk food consumption by 9 months.

	Unadjusted		Adjusted – Full Model^†^		Adjusted - Reduced model^‡^	
Characteristics	Relative Risks (95% CI)	*p* value	Relative Risks (95% CI)	*p* value	Relative Risks (95% CI)	*p* value
Site						
DRC	REF		REF		REF	
Zambia	8.84 (4.65,16.79)	<0.01	9.18 (4.60,18.32)	<0.01	9.08 (4.89,16.86)	<0.01
Guatemala	6.78 (3.55,12.94)	<0.01	6.25 (3.12,12.54)	<0.01	6.95 (3.72,12.96)	<0.01
Pakistan	9.12 (4.79,17.33)	<0.01	8.03 (4.02,16.03)	<0.01	9.24 (4.97,17.18)	<0.01
Birthweight	0.98 (0.89,1.09)	0.75	1.00 (0.91,1.11)	0.95	---	---
Female	1.03 (0.93,1.14)	0.54	0.99 (0.88,1.11)	0.89	---	---
Exclusively breastfed at 6 months	0.83 (0.68,1.00)	0.05	0.90 (0.70,1.17)	0.43	---	---
Prematurity	1.14 (0.91,1.41)	0.25	1.15 (0.93,1.44)	0.20	---	---
Vitamin supplements	1.11 (0.94,1.32)	0.21	0.94 (0.76,1.15)	0.52	---	---
Number of living children						
1–2	1.02 (0.87,1.20)	0.77	---	---	1.00 (0.85,1.17)	0.96
3–4	0.89 (0.75,1.06)	0.20	---	---	0.88 (0.75,1.04)	0.13
≥5	REF		---	---	REF	
Maternal BMI	0.99 (0.97,1.01)	0.41	---	---	0.99 (0.98,1.01)	0.22
DRC	---	---	0.97 (0.80, 1.18)	0.78	---	---
Zambia	---	---	1.01 (0.99, 1.03)	0.34	---	---
Guatemala	---	---	0.97 (0.95, 1.00)	0.05	---	---
Pakistan	---	---	0.98 (0.97, 0.99)	<0.01	---	---
Maternal education						
0	1.00 (0.83,1.19)	0.96	0.99 (0.81,1.22)	0.95	---	---
1–7	1.03 (0.87,1.22)	0.74	1.08 (0.92,1.26)	0.34	---	---
≥8	REF		REF		---	---
Paternal education						
0	1.01 (0.85,1.18)	0.95	0.98 (0.78,1.21)	0.82	---	---
1–7	1.00 (0.91,1.11)	0.95	0.99 (0.88,1.12)	0.87	---	---
≥8	REF		REF		---	---
SES						
Low (≤−1.3)	0.74 (0.51,1.09)		1.01 (0.77,1.33)	0.95	---	---
Medium (−1.3 < SES < 2.8)	1.05 (0.92,1.19)		1.06 (0.92,1.21)	0.43	---	---
High (≥2.8)	REF		REF		---	---

^†^Relative risks were estimated using a Poisson regression model with robust variance estimators and included potential predictors of birthweight, sex, exclusively breastfed at 6 months, prematurity, vitamin supplements, number of living children, maternal BMI, maternal education, paternal education, and SES (Socioeconomic Status) while controlling for site (DRC, Zambia, Guatemala, Pakistan) and intervention. Tests of whether the association between each potential predictor and the outcome differed by site were conducted. Interactions with site were significant for maternal BMI (*p* = 0.04); adjusted relative risks are shown by site for these measures.

^‡^Stepwise selection was used to narrow down the number of potential predictors in the model. A significance level of 0.30 was required to allow a variable into the model, and a significance level of 0.35 was required for the variable to stay in the model. Relative risks were estimated using a Poisson regression model with robust variance estimators and included the following potential predictors after stepwise selection: number of living children, maternal BMI, site and intervention. Tests of whether the association between each potential predictor and the outcome differed by site were conducted; none were significant.

**Table 5:** Potential Predictors for Junk Food Consumption by 9 Months without DRC.

	Unadjusted		Adjusted – Full model^†^		Adjusted – Reduced Model^‡^	
Characteristics	Relative Risks (95% CI)	*p* value	Relative Risks (95% CI)	*p* value	Relative Risks (95% CI)	*p* value
Site						
Zambia	0.97 (0.84,1.12)	0.68	1.21 (0.79,1.85)	0.39	1.12 (0.84,1.49)	0.44
Guatemala	0.74 (0.63,0.87)	<0.01	0.79 (0.63,0.98)	0.04	0.78 (0.66,0.91)	<0.01
Pakistan	REF		REF		REF	
Birthweight	0.98 (0.89,1.08)	0.72	0.99 (0.89,1.09)	0.78	---	---
Female	1.01 (0.91,1.11)	0.92	0.96 (0.87,1.07)	0.49	---	---
Exclusively breastfed at 6 months	0.99 (0.88,1.12)	0.89	0.86 (0.65,1.12)	0.26	0.88 (0.68,1.13)	0.30
Prematurity	1.12 (0.94,1.33)	0.22	---	---	---	---
Zambia	---	---	1.09 (0.83,1.42)	0.53	1.12 (0.87,1.43)	0.39
Guatemala	---	---	1.58 (1.33,1.88)	<0.01	1.56 (1.33,1.83)	<0.01
Pakistan	---	---	0.85 (0.59,1.23)	0.38	0.87 (0.70,1.09)	0.24
Vitamin supplements	1.00 (0.90,1.11)	0.99	0.94 (0.77,1.15)	0.55	---	---
Number of living childre						
1–2	0.96 (0.82,1.12)	0.62	0.99 (0.80,1.21)	0.89	0.94 (0.80,1.11)	0.48
3–4	0.88 (0.76,1.03)	0.11	0.84 (0.70,1.03)	0.09	0.87 (0.75,1.02)	0.08
≥5	REF		REF		REF	
Maternal BMI	0.99 (0.97,1.00)	0.16	---	---	0.99 (0.97,1.00)	0.17
Zambia	---	---	1.01 (0.99, 1.03)	0.36	---	---
Guatemala	---	---	0.97 (0.95, 1.00)	0.04	---	---
Pakistan	---	---	0.98 (0.97, 0.99)	<0.01	---	---
Maternal education						
0	1.10 (0.97,1.24)	0.13	1.06 (0.89,1.26)	0.53	---	---
1–7	1.00 (0.87,1.15)	0.99	1.05 (0.89,1.22)	0.58	---	---
≥8	REF		REF		---	---
Paternal education						
0	1.04 (0.91,1.19)	0.57	1.01 (0.81,1.25)	0.94	---	---
1–7	1.01 (0.91,1.12)	0.90	1.01 (0.89,1.14)	0.89	---	---
≥8	REF		REF		---	---
SES						
Low (≤−1.3)	1.06 (0.82,1.37)	0.66	1.00 (0.75,1.33)	1.00	---	---
Medium (−1.3 < SES < 2.8)	1.07 (0.96,1.20)	0.21	1.04 (0.91,1.19)	0.58	---	---
High (≥2.8)	REF		REF		---	---

^†^Relative risks were estimated using a Poisson regression model with robust variance estimators and including potential predictors of birthweight, sex, exclusively breastfed at 6 months, prematurity, vitamin supplements, number of living children, maternal BMI, maternal education, paternal education, and SES (Socioeconomic Status) while controlling for site (Zambia, Guatemala, Pakistan) and intervention. Tests of whether the association between each potential predictor and the outcome differed by site were conducted. Interactions with site were significant for prematurity (*p* < 0.01) and maternal BMI (*p* = 0.01); adjusted relative risks are shown by site for these measures.

^‡^Stepwise selection was used to narrow down the number of potential predictors in the model. A significance level of 0.30 was required to allow a variable into the model, and a significance level of 0.35 was required for the variable to stay in the model. Relative risks were estimated using a Poisson regression model with robust variance estimators and included the following potential predictors after stepwise selection: exclusively breastfed at 6 months, prematurity, number of living children, maternal BMI, site, and intervention. Tests of whether the association between each potential predictor and the outcome differed by site were conducted. Interactions with site were significant for prematurity (*p* < 0.01); adjusted relative risks are shown by site for these measures.

Overall, consumption of any junk food, candy, salty snacks, and sweet drinks was not associated with adverse Mental Developmental Index (MDI) or Psychomotor (PDI) scores (Table 6). Infants who were not exclusively breastfed up to 6 months showed similar adjusted mean Mental Developmental Index (MDI) scores as those who were exclusively breastfed (93.6 vs. 92.0, *p* = 0.24). However, infants who were not exclusively breastfed up to 6 months showed a higher adjusted mean Psychomotor Developmental Index (PDI) score at 18 months than those who were exclusively breastfed (99.8 vs. 95.2, *p* = 0.02). Because in Zambia, cookie consumption by 12 months was associated with higher adjusted mean PDI scores compared to infants who did not consume cookies (104.5 vs. 101.0, *p* = 0.01), MDI and PDI results by country are also included (Table 6). Socioeconomic status was not associated with adverse MDI or PDI scores (Table 6). Prior intake of junk food at any age was not associated with anthropometric measures (length, weight, weight-for-length, or head circumference) (Supplementary Tables S1, S2).

**Table 6:** Unadjusted and adjusted means for Bayley Scales of Infant Development measures at 18 months.

			Mental Developmental Index (MDI)			Physical Developmental Index (PDI)		
Measures		*N* ^†^	Mean ± SD (unadjusted)	Mean (adjusted)^‡^	*p* value^‡^	Mean ± SD (unadjusted)	Mean (adjusted)^‡^	*p* value^‡^
Exclusively breastfed at 6 months	Yes	269	97.74 ± 10.61	92.02	0.24	100.55 ± 11.12	96.25	0.02
	No	495	93.38 ± 9.14	93.62		99.37 ± 10.27	99.78	
SES tercile	Low	81	97.51 ± 11.11	91.39	0.20	100.70 ± 10.53	96.60	0.24
	Medium	443	94.75 ± 10.28	92.92		99.39 ± 11.06	98.07	
	High	240	94.34 ± 8.57	94.14		100.20 ± 9.68	99.37	
Cookies								
9 months	Yes	325	95.24 ± 10.06	93.48	0.15	100.77 ± 10.14	98.95	0.06
	No	439	94.67 ± 9.78	92.35		99.05 ± 10.85	97.35	
12 months	Yes	489	95.26 ± 10.08	93.37	0.06	100.32 ± 10.53	---	---
	No	275	94.29 ± 9.55	91.88		98.84 ± 10.64	---	
Zambia	Yes	131	---	---	---	103.51 ± 9.41	104.50	0.01
	No	100	---	---		100.49 ± 10.46	100.96	
Guatemala	Yes	130	---	---	---	95.59 ± 12.24	92.36	0.33
	No	133	---	---		96.79 ± 11.30	93.59	
Pakistan	Yes	228	---	---	---	101.18 ± 9.11	98.24	0.99
	No	42	---	---		101.38 ± 7.37	98.23	
Candy								
9 months	Yes	43	97.14 ± 10.67	94.28	0.33	100.21 ± 9.95	98.43	0.80
	No	721	94.78 ± 9.84	92.77		99.76 ± 10.63	98.00	
12 months	Yes	105	95.13 ± 9.39	92.74	0.93	99.07 ± 10.82	97.27	0.46
	No	659	94.88 ± 9.98	92.83		99.90 ± 10.55	98.14	
Salty snacks								
9 months	Yes	181	95.46 ± 10.61	91.65	0.10	100.93 ± 10.85	97.50	0.51
	No	583	94.74 ± 9.66	93.20		99.43 ± 10.49	98.17	
12 months	Yes	346	95.71 ± 10.34	92.49	0.45	100.86 ± 9.94	97.86	0.75
	No	418	94.24 ± 9.47	93.13		98.89 ± 11.02	98.15	
Sweet drinks								
9 months	Yes	210	96.07 ± 9.94	93.1	0.63	99.04 ± 11.64	97.48	0.39
	No	554	94.48 ± 9.85	92.69		100.07 ± 10.15	98.27	
12 months	Yes	367	95.42 ± 10.19	92.66	0.68	99.12 ± 10.91	97.82	0.64
	No	397	94.45 ± 9.61	93.01		100.40 ± 10.25	98.24	
Junk food								
9 months	Yes	501	95.09 ± 9.87	92.91	0.70	100.13 ± 10.69	98.22	0.45
	No	263	94.57 ± 9.96	92.62		99.14 ± 10.36	97.59	
12 months	Yes	677	95.02 ± 10.02	92.85	0.71	99.89 ± 10.57	98.02	0.94
	No	87	94.01 ± 8.87	92.42		98.98 ± 10.70	97.92	

^†^Numbers are among the 1,042 children with non-missing BSID-II scores. BSID-II scores were missing for 17/1062 (1.6%) children [Zambia: 12/246 (4.9%); Guatemala: 3/267 (1.1%); Pakistan: 2/278 (1.0%)]. SES (Socioeconomic Status).

^‡^BSID-II means were estimated using linear models fit to each Bayley index score. Models for each junk food type adjust for SES, site (Zambia, Guatemala, Pakistan), intervention group, sex, exclusively breastfed first 6 months, birth weight, preterm status, vitamin supplementation, maternal BMI, maternal education level, and number of living children. Tests of whether the association between measure and outcome differed by site were conducted. Interaction with site was significant for cookie consumption at 12 months (*p* = 0.04); PDI score means are shown by site for this measure. The models for exclusively breastfed first 6 months and SES only adjust all factors excluding junk food intake.

## 4 Discussion

The study sought to determine the predictors of feeding junk foods to infants and toddlers aged 6 to 18 months in low-resource countries. Junk food feeding was lower in DRC than in the other countries. After removal of the DRC data, the baseline characteristics did not differ between infants feed junk foods and those not feed junk food. Overall, the rate of feeding junk food was relatively high (7% at 6 months, 51% at 9 months, 70% at 12 months, and 78% at 18 months).

A strength of this study was the inclusion of infants in diverse, low-resource settings on three continents with data collected retro-prospectively. However, the data do not necessarily represent broader regions from the countries in which the clusters were located. In addition, the high prevalence use rate of junk foods occurred even though mothers in the trial were given appropriate nutritional and health promotion messages on the importance of exclusive breastfeeding up to 6 months with continued breastfeeding up to at least 12 months. The demographic data and the data on junk foods fed to infants were obtained prospectively from mothers’ and/or caretakers’ reports so bias should have been minimized. An important limitation is that we did not record product details or the nutritional values of the specific foods considered junk food including the protein or micronutrient content in relation to calories, the proportion of calories as sugar or fat, or the amount of salt. Furthermore, the qualitative nature of the tool did not allow for quantitative estimates of junk food intake. However, we prospectively defined the categories of food considered generally to be junk foods.

The current study discovered that feeding junk foods varied widely between countries despite the availability of study foods and health messages on quality and quantity of complementary foods. The DRC communities, where junk foods were fed least frequently, were the most remote and least commercialized with the least availability of junk food for purchase. Thus, limited access and poverty were likely critical factors. Reviews done in low-resource settings have found that infants are fed on available foods regardless of their nutrition value (9, 24). Feeding infants in these countries can be linked to availability and exposure to junk foods. Parents are the role models for the infants and toddlers in choice of food and feeding patterns and likely feed their infants on available foods as observed in this study (25–27).

The current study revealed that 7% of the infants were fed junk foods at the age of 6 months, and this increased to 70% at 18 months. Feeding junk food at 6 months also occurred in a study conducted in rural parts of Zambia, which reported that caregivers knew that breastfeeding was sufficient up to 6 months, but still gave complementary food of inadequate quality before 6 months (28). Similar studies conducted in Egypt, which assessed early introduction of complementary feeding and associated factors, observed that infants were fed on sweetened foods and drinks based on the knowledge, practice, and cultural beliefs of the mothers; they reported that 28% of 120 infants less than 2 years of age were given junk food based on the mothers’ perceptions (29), which is lower that what we found. Reviews from developing countries noted that consumption of junk food was increasing as countries traverse the “nutrition transition” (25, 30). Noteworthy, developed countries have not been spared, implying that it is not so much limited resources but attitude, lifestyle, cultural practices, and availability of less nutritious foods (31). Food containing low nutritional value likely displaces potentially higher quality foods and thus deprives infants of the much-needed nutrients (32).

Studies conducted in Ethiopia and Senegal have concluded that inappropriate complementary feeding are associated with adverse children’s development and growth (33, 34). Inappropriate complementary feeds were given to 91.5% of 6 to 23 months children of 611 mothers surveyed in Ethiopia (33) and 93.5% of 6 to 23 months children of 293 mothers surveyed in Senegal (33)., which is comparable to the rates we observed in DRC, Guatemala, and Zambia at 18 months. However, despite similar rates of junk food intake, the current study did not confirm the associations of junk food intake and children’s development and growth in these or other studies (35, 36) that show that inappropriate feeding practices during infancy may hinder the development or growth. The lack of an association of inappropriate complementary feeds as part of the trial could be due to the high quality of the complementary feeds given to both the intervention and control group infants.

The data on junk food intake is limited to association studies, which have limited value for robust results. However, some randomized controlled trials have shown that some neurodevelopmental improvements with interventions of high nutritional value. In a randomized controlled trial of supplemental protein intake and micronutrient intake in India that included over 2,000 infants, motor scores were improved by a mean of 1.52 points (95% CI: 0·28, 2·75) at 12 months, there were no differences in developmental scores at 24 month (37). However, a trial of zinc, iron, and copper supplementation starting at 6 months in 251 infants in Peru with Bayley assessments at 18 months reported no significant neurodevelopmental benefits (38). Furthermore, a meta-analysis of 21 trials and 14,241 infants conducted mostly middle- and low-income countries showed that nutrition education related to weaning from breast feeding modestly improved weight gain and height by 12 months but inconsistent effects were observed at 18 months of age. Neurodevelopmental outcomes were not assessed as none of the 21 trials included those assessments (39).

This secondary study had important limitations. Detailed data collection on junk or processed foods were not obtained. The exposure and specific contents of the junk foods were limited to any use at defined time periods and qualitative 24-h recall was qualitative rather than quantitative analysis of energy, fat, sugar, and salt or the degree the processed food were processed. The quality of nutritional data collected based on 24-h recall is limited. We did not count caloric intake from junk food but a recent study from Nepal of 745 children under 2 years of age that also reported high rates of junk food intake (91%) comparable to our present report, found that the proportion of total energy intake of non-breast milk intake was 24.5% among all children (40) indicating the nutritional value of the total intake can be adversely affected.

## 5 Conclusion

This prospective study on nutrition in low-resource settings in several countries revealed that the practice of feeding low nutritional value foods was widespread in the study sites but varied by setting. These findings occurred despite nutritional and health promotion messages on the importance of exclusive breastfeeding and the need for nutritious food from 6 to 18 months given to the mothers and caretakers. In this study, non-exclusive breastfeeding at 6 months, prematurity, higher maternal body mass index, more years of maternal and paternal education, and higher socioeconomic scores were associated with more consumption of junk food at 9 and 12 months. Data collection on food intake was qualitative without nutritional quantification. Prevalent use of junk foods was not associated with adverse neurodevelopmental or growth outcomes, but all participants received nutritious complementary feeds including daily meat or fortified cereal complementary food from 6 to 18 months and nutritious feeds messages as part of a nutrition trial. The impact of such frequent consumption of junk foods during this critical period development might differ if they displace nutritious foods. Health promotion messages should attempt to encourage parents to feed infants appropriate complementary feeds.

## Data availability statement

The data analyzed in this study is subject to the following licenses/restrictions: the data used in this analysis is available through NICHD Data and Specimen Hub (N-DASH). Requests to access these datasets should be directed to https://dash.nichd.nih.gov/.

## Ethics statement

This research study was reviewed for approval by the Institutional Review Board (IRB) of each participating institution. Annual reviews are conducted as per each institution's standard review procedures. The IRBs are registered at OHRP with FWAs. Safety and efficacy analyses are performed at scheduled intervals based upon age (after every 100 infants reached one year of age) and the completion of study participation. Written informed consent for trial participation was obtained from the women, or the parents or guardians of minors, by trained staff.

## Author contributions

MC: Conceptualization, Methodology, Writing – review & editing. NK: Conceptualization, Formal analysis, Investigation, Methodology, Supervision, Writing – original draft. AM: Supervision, Writing – review & editing. EC: Conceptualization, Investigation, Writing – review & editing. MMw: Investigation, Writing – review & editing. MMa: Investigation, Writing – review & editing. NS: Investigation, Writing – review & editing. OP: Investigation, Writing – review & editing. AT: Investigation, Supervision, Writing – review & editing. AL: Investigation, Writing – review & editing. RG: Investigation, Supervision, Writing – review & editing. CB: Investigation, Supervision, Writing – review & editing. MK-T: Writing – review & editing. NG: Formal analysis, Writing – review & editing. BD: Formal analysis, Writing – review & editing. EM: Formal analysis, Writing – review & editing. KH: Investigation, Writing – review & editing. JW: Investigation, Writing – review & editing. WC: Conceptualization, Investigation, Writing – original draft, Writing – review & editing.
