# Supplementary material for: Junk food use and neurodevelopmental and growth outcomes in infants in low-resource settings
Source: Front Public Health. 2024 Apr 15;12:1308685. doi: 10.3389/fpubh.2024.1308685 (PMC11057493; doi:10.3389/fpubh.2024.1308685)
Supplement: Supplementary file 1 [file Data_Sheet_1.PDF]

Table S1. Unadjusted and Adjusted Means for 18-month Anthropometric Measures

Measures		N‡	Length Z-Score†			Weight Z-Score†			Weight-for-Length Z-Score†		
			Mean ± SD (unadjusted)	Mean (adjusted)§	P value§	Mean ± SD (unadjusted)	Mean (adjusted)§	P value§	Mean ± SD (unadjusted)	Mean (adjusted)§	P value§
Exclusively breastfed	Yes	275	-1.74 ± 1.31	-2.23	0.59	-0.89 ± 1.04	-1.38	0.78	-0.07 ± 1.07	-0.38	0.97
at 6 months	No	487	-2.02 ± 1.22	-2.16		-1.42 ± 0.99	-1.35		-0.6 ± 1.13	-0.39	
SES tercile	Low	82	-1.72 ± 1.31	-2.38	0.03	-0.95 ± 1.09	-1.50	0.06	-0.17 ± 1.28	-0.43	0.67
	Medium	447	-1.99 ± 1.31	-2.22		-1.29 ± 1.05	-1.40		-0.43 ± 1.13	-0.41	
	High	233	-1.85 ± 1.14	-1.98		-1.21 ± 1.00	-1.20		-0.45 ± 1.10	-0.32	
Cookies											
9 months	Yes	314	-1.76 ± 1.21	-2.17	0.57	-1.34 ± 1.05	-1.38	0.66	-0.67 ± 1.18	-0.44	0.27
	No	448	-2.03 ± 1.29	-2.21		-1.15 ± 1.03	-1.35		-0.23 ± 1.07	-0.35	
12 months	Yes	480	-1.81 ± 1.23	-2.18	0.62	-1.31 ± 1.05	-1.39	0.32	-0.60 ± 1.17	-0.44	0.10
	No	282	-2.11 ± 1.28	-2.22		-1.09 ± 1.01	-1.32		-0.09 ± 1.01	-0.30	
Candy											
9 months	Yes	45	-1.73 ± 1.34	-1.95	0.13	-1.06 ± 1.06	-1.40	0.79	-0.29 ± 1.02	-0.59	0.18
	No	717	-1.93 ± 1.26	-2.20		-1.24 ± 1.04	-1.36		-0.42 ± 1.15	-0.38	
12 months	Yes	106	-1.79 ± 1.20	-1.99	0.04	-1.17 ± 1.07	-1.40	0.67	-0.38 ± 1.02	-0.55	0.09
	No	656	-1.94 ± 1.27	-2.23		-1.24 ± 1.04	-1.36		-0.41 ± 1.16	-0.36	
Salty snacks											
9 months	Yes	184	-1.55 ± 1.15	-2.17	0.74	-1.04 ± 0.98	-1.32	0.56	-0.41 ± 1.21	-0.35	0.62
	No	578	-2.04 ± 1.27	-2.20		-1.29 ± 1.05	-1.38		-0.41 ± 1.12	-0.40	
12 months	Yes	346	-1.59 ± 1.18	-2.16	0.46	-1.06 ± 1.02	-1.32	0.32	-0.41 ± 1.19	-0.36	0.52
	No	416	-2.19 ± 1.26	-2.23		-1.37 ± 1.04	-1.40		-0.41 ± 1.09	-0.41	
Zambia	Yes	174	---	---	---	-0.68 ± 0.88	-0.63	0.02	---	---	---
	No	63	---	---		-0.96 ± 1.13	-0.97		---	---	

Guatemala	Yes	37	---	---	---	-1.29 ± 1.13	-1.40	0.39	---	---	---
	No	230	---	---		-1.38 ± 0.96	-1.54		---	---	
Pakistan	Yes	135	---	---	---	-1.50 ± 0.97	-1.92	0.13	---	---	---
	No	125	---	---		-1.55 ± 1.08	-1.70		---	---	
Sweet drinks											
9 months	Yes	212	-2.03 ± 1.27	-2.15	0.51	-1.01 ± 0.97	-1.28	0.14	-0.04 ± 0.96	-0.31	0.18
	No	550	-1.88 ± 1.25	-2.21		-1.32 ± 1.06	-1.40		-0.55 ± 1.17	-0.42	
12 months	Yes	365	-2.05 ± 1.29	-2.11	0.06	-1.04 ± 0.98	-1.27	0.02	-0.07 ± 0.95	-0.32	0.12
	No	397	-1.80 ± 1.22	-2.29		-1.40 ± 1.06	-1.47		-0.72 ± 1.21	-0.46	
Junk food											
9 months	Yes	496	-1.86 ± 1.23	-2.19	0.98	-1.25 ± 1.02	-1.39	0.35	-0.49 ± 1.15	-0.42	0.16
	No	266	-2.03 ± 1.31	-2.20		-1.18 ± 1.08	-1.32		-0.26 ± 1.10	-0.31	
12 months	Yes	673	-1.88 ± 1.24	-2.19	0.58	-1.24 ± 1.04	-1.37	0.87	-0.44 ± 1.14	-0.39	0.50
	No	89	-2.21 ± 1.34	-2.26		-1.18 ± 1.07	-1.35		-0.14 ± 1.07	-0.31	

†Z-scores deemed implausible according to WHO criteria were set to missing. This includes: WAZ < -6SD or > 5SD; LAZ < -6SD or >6SD; WHZ < -5SD or >5SD.
‡N includes those with non-missing measurements.
§Adjusted means were estimated using linear models fitting each anthropometric outcome. Models for each junk food type adjust for SES, site (Zambia, Guatemala, Pakistan), intervention group, sex, exclusively breastfed first 6 months, birth weight, preterm status, vitamin supplementation, maternal BMI, maternal education level, and number of living children. Tests of whether the association between each measure and outcome differed by site were conducted. Interaction with site was significant for salty consumption by 12 months (p=0.02); WAZ scores are shown by site for this measure. The models for exclusively breastfed first 6 months and SES only adjust all factors excluding junk food intake.

Table S2. Unadjusted and adjusted means for head circumference Z-scores at 18 months

Measures		N[‡]	Head Circumference Z-Score[†] Mean ± SD (unadjusted)	Mean (adjusted)[§]	P value[§]
Exclusively breastfed at 6 months	Yes	284	-0.39 ± 0.97	-0.76	0.83
	No	506	-0.83 ± 0.97	-0.79	
SES tercile	Low	83	-0.40 ± 1.10	-0.78	0.78
	Medium	465	-0.68 ± 0.97	-0.80	
	High	242	-0.74 ± 0.99	-0.74	
Cookies					
9 months	Yes	332	-0.72 ± 1.08	-0.82	0.26
	No	458	-0.63 ± 0.92	-0.73	
12 months	Yes	503	-0.68 ± 1.04	-0.76	0.75
	No	287	-0.64 ± 0.90	-0.79	
Candy					
9 months	Yes	46	-0.60 ± 1.16	-0.82	0.76
	No	744	-0.67 ± 0.98	-0.77	
12 months	Yes	109	-0.62 ± 1.14	-0.76	0.87
	No	681	-0.68 ± 0.97	-0.77	
Salty snacks					
9 months	Yes	193	-0.53 ± 1.04	-0.77	0.98
	No	597	-0.71 ± 0.97	-0.77	
12 months	Yes	363	-0.56 ± 0.95	-0.78	0.81
	No	427	-0.76 ± 1.02	-0.76	
Sweet drinks					
9 months	Yes	216	-0.58 ± 0.98	-0.76	0.86
	No	574	-0.70 ± 1.00	-0.78	
12 months	Yes	376	-0.55 ± 0.95	-0.69	0.04
	No	414	-0.78 ± 1.02	-0.86	
Junk food					
9 months	Yes	517	-0.69 ± 1.02	-0.81	0.17
	No	273	-0.64 ± 0.93	-0.70	
12 months	Yes	699	-0.67 ± 1.00	-0.77	0.82
	No	91	-0.67 ± 0.94	-0.75	

[†] Z-scores deemed implausible (<-5SD or >5SD) according to WHO criteria were set to missing.
[‡] N includes those with non-missing measurements.
[§] BSID-II means were estimated using linear models fit to each Bayley index score. Models for each junk food type adjust for SES, site (Zambia, Guatemala, Pakistan), intervention group, sex, exclusively breastfed first 6 months, birth weight, preterm status, vitamin supplementation, maternal BMI, maternal education level, and number of living children. The models for exclusively breastfed first 6 months and SES only adjust all factors excluding junk food intake.
